# Designing a core data set for benign hysterectomy registration system and its implementation in a referral teaching hospital in Northwest Iran

**DOI:** 10.1186/s12884-024-06656-z

**Published:** 2024-07-03

**Authors:** Fatemeh Moghadami Asl, Elham Maserat, Maryam Vaezi, Zeinab Mohammadzadeh

**Affiliations:** 1 Department of Health Information Technology, School of Management and Medical Informatics, Tabriz University of Medical Sciences, Daneshgah St, Tabriz, 5165665811 Iran; 2 Department of Medical Informatics, Faculty of Medical Sciences, Tarbiat Modares University, Tehran, Iran; 3 Associate professor of Gynecology, Oncology, Department of Obstetrics and Gynecology, Women’s Reproductive Health Research Center, Clinical Research Institute, Alzahra Hospital, Tabriz University of Medical Sciences, Tabriz, Iran

**Keywords:** Hysterectomy, Registry, Minimum data set, Patient registry, Minimally invasive hysterectomy

## Abstract

**Background and aims:**

Although minimally invasive hysterectomy offers advantages, abdominal hysterectomy remains the predominant surgical method. Creating a standardized dataset and establishing a hysterectomy registry system present opportunities for early interventions in reducing volume and selecting benign hysterectomy methods. This research aims to develop a dataset for designing benign hysterectomy registration system.

**Methods:**

Between April and September 2020, a qualitative study was carried out to create a data set for enrolling patients who were candidate for hysterectomy. At this stage, the research team conducted an information needs assessment, relevant data element identification, registry software development, and field testing; Subsequently, a web-based application was designed. In June 2023the registry software was evaluated using data extracted from medical records of patients admitted at Al-Zahra Hospital in Tabriz, Iran.

**Results:**

During two months, 40 patients with benign hysterectomy were successfully registered. The final dataset for the hysterectomy patient registry comprise 11 main groups, 27 subclasses, and a total of 91 Data elements. Mandatory data and essential reports were defined. Furthermore, a web-based registry system designed and evaluated based on data set and various scenarios.

**Conclusion:**

Creating a hysterectomy registration system is the initial stride toward identifying and registering hysterectomy candidate patients. this system capture information about the procedure techniques, and associated complications. In Iran, this registry can serve as a valuable resource for assessing the quality of care delivered and the distribution of clinical measures.

## Introduction

Hysterectomy is one of the common surgical procedures for gynecological diseases following cesarean section, is performed in over 80% of cases to treat of benign uterus diseases such as leiomyoma, abnormal uterine bleeding, pelvic organ prolapses, endometriosis, abdominal pain and prevention of future malignancy [1–3]. Hysterectomy can be performed using various approaches including abdominal, vaginal, or laparoscopic methods, with or without robotic assistance [4–6].

According to the Cochrane study, vaginal hysterectomy is the most effective approch for a quick return to normal activities and early hospital discharge compared to laparoscopic and abdominal hysterectomy. As a result, it is considered the superior approach for hysterectomy, particularly, for benign diseases, where minimally invasive procedures are preferred [4].

As per the recommendation of American College of Obstetricians and Gynecologists and the American Society of Laparoscopic Gynecologists, minimally invasive hysterectomy (MIS) is the performed approach whenever feasible [7]. because it offers well-established benefits compared to abdominal hysterectomy. Unless it is not possible due to the characteristics of the patient, disease or technically [8].

Despite well documented benefits of minimally invasive hysterectomy (MIS) [4, 7], there is significant variation in hysterectomy rates and procedures across countries. [9, 10]. These variations can reflect discord in characteristics, resource allocation, and medical personnel recruitment, experience, or education [11].

The evidence of hysterectomies in Iran is mostly limited due to a lack of sufficient information in extensive national health surveys [12, 13]. Therefore, it is important to determine the trend and rate of hysterectomies [7]. Administrative data collected to determine the frequency and complications of hysterectomy is limited Applicability [14]. Creating a patient registry system is a valuable method for systematic data collection [15]. Eddentially the term “patient registry” refers to the organized recording of health information from different sources and documents. The World Health Organization defines registries in medical information systems as “document files containing unified information about individuals, collected systematically or exhaustively for later use with definite purposes” [16]. Considering the influense of technology and training on hysterectomy method is crucial [7]. Establishing a registry to monitor hysterectomy procedures can enhance healthcare professionals’ education and knowledge in this area [14].

Creating a minimum data set (MDS) is a foundational steps to ensure standardized data collection in disease registries [17, 18]. Developing a standardized dataset for clinical registries provides better use of health information and enhances the quality of medical care [19]. Additionally, MDS creates a uniform approach to health information management by defining and standardizing essential data elements for a specific disease [15, 20]. This objective of this study is to design and implement a registration system for ongoing monitoring hysterectomy trends. The database will track the proportion of hysterectomies performed by different routes including abdominal, vaginal, and laparoscopic. Also it will analyze clinical and demographic factors associated with undergoing hysterectomy at different levels.

## Methods

To create a data set for the hysterectomy patient registry, a qualitative Delphi method was employed using multiple rounds of data collection conducted between March and June of 2021. This stage included information needs assessment, data elements identification, registry software development, and field-testing the system.

### Information needs assessment

After reviewing the relevant research topics and existing similar national and international registration systems, also interviews with experts in the field of gynecology the content analysis method was employed to prepare core data elements. Content analysis is an effective approach to determine the presence of specific words, themes, or concepts in qualitative data such as literature [21].

### Data elements identification

During this phase of study, the comprehensive search was conducted to gather relevant information across various database and search engine including PubMed, Embase, Scopus, Cochrane, and Google Scholar. In addition, we searched Iranian databases including Irandoc, SID, and Magiran to retrieve articles in the Persian language. “Dataset”, “MDS”, “minimum data set”, “registry”, “hysterectomy”, “patient registry” and “Database” and their Subject Headings (MeSH) were the keywords used in conducting the search strategy. Except for hysterectomy forms, only publications in Persian and English languages published between May 2000 and May 2021 were included. Letters to editors and reports retrieved from websites were excluded. All related data elements of the final selected full texts were recorded for use in the Delphi questionnaire. The expert panel method was then employed used to select the data set. This method is based on the consensus and alignment of experts on the study subject and question. This method has been used in a large number of studies to determine the minimum data set that requires agreement on important data elements [22, 23].

In this study Expert panel members included two Gynecological oncologists, one assistant professor of laparoscopic surgery, one urogynecologist, one Infertility Fellowship, and two specialists in Health Information Management. In the next phase, we developed a semi-structured electronic questionnaire containing a comprehensive list of data elements to identify MDS. Also expert opinions were collected and applied in the final checklist [24]. Additionally, the expert panel specified which data were mandatory and which were optional. After determining the data set for the registry, a data dictionary was prepared for design of the registration software. Subsequently inclusion and exclusion criteria were defined. Exclusion criteria were age over 65 years, emergency hysterectomy, and malignant hysterectomy.

### Registry software development

In the next step, the hysterectomy patient registration software was designed to collect data from health centers using the model sampling method. The prototype model was employed a software development model in which a prototype is iteratively constructed and tested until a satisfactory version is achieved [16, 23].

The hysterectomy registration software is a web-based application developed using Visual Studio2019. The main framework is ASP.NET MVC, with C# as the development language. The data is stored in a SQL Server database. The Hysterectomy registration software was shared with three members of the project team at http://irhyst.ir for testing and consulting. Then to finalize and adress problems from the initial version of the software, several meetings were held conducted with the software design team. In the next step, the identified mandatory data set in the software, and its completion was required to answer the questions on the next pages. Additionally, the registry dashboard dynamically provided important reports as requested by Physicians, allowing them to select the desired time period.

### Field testing system

After the software preparation, the registration program was run as a pilot. Al-Zahra Hospital, a prominent referral teaching hospital in northwestern Iran (located in Tabriz city), was chosen for this initial implementation. During the pilot implementation of the registry program for three weeks, several changes in values and ambiguous questions were made. This modifications included refining values, clarifying ambiguous questions, adding or removing data elements, adjusting titles, and fine-tuning the values associated with specific elements.

After the pilot step, in the second phase, we will initiate implementing a hysterectomy patient registry in health centers of Tabriz City from August 2023.

## Results

As part of system requirements, the team of experts prioritized the need to record and report on diverse hysterectomy procedures, along with their respective percentage distributions. Additionally, the system should capture patient age averages, primary reasons for hysterectomy, and common pathology findings. The system design should allow for the creation of printed reports and the retrieval of system outputs, as requested by the experts. All these essential performance metrics have been seamlessly integrated into the system dashboard.

In the information sources review step, a total of 101 data elements were identified across 22 subclasses and 11 main classes. Based on the expert panel opinion, we removed 12 data elements and added 2 new data elements (for evaluating interventions). Unnecessary values for certain elements, such as Urinary incontinence, Surgery turn, Surgeon’s medical system number, and Surgeon Assistant System Number, have been removed. Some data elements were added to the data classes such as suspension and incisions. Here are the adjustments made to enhance data quality:

- Bleeding rate and uterine weight have been changed from mandatory to optional due to the unavailability of information.
- To maintain data quality, we have set minimum and maximum data limits for quantitative variables, preventing the entry of outliers.

The final data set for the hysterectomy patient registry comprises 11 main groups, 27 subclasses, and 91 data elements (as detailed in Table 1). The 26 administrative data elements, were classified into 5 categories: Demographic data, Socioeconomic, address, Patient ADT, and registrant relate data (Table 2).

**Table 1:** Data set of hysterectomy patient registry

Main groups	The number of main classes	The number of subclasses	The number of the data elements
Administrative data	3	6	26
Patient History	1	3	13
Operation data	2	7	18
Additional interventions	2	5	16
Perioperative complications	1	3	9
Follow up data	2	3	9
Total	11	27	91

**Table 2:** Administration data

Main class	Subclass	Mandatory/ Optional data	Data elements	The Number of data elements
patient Profile	Demographic data	M M	National identity number, Patient name, Patient surname, Date of birth, marital status	5
	Socio-economic	O M	Educational degree, Job title (Employment status), Type of residence (urban, suburban, rural)	3
	address	M	Mobile phone number, Telephone number, address details	3
Hospital profile	specifications Patient ADT	M M O M M M 0	Healthcare center name, Patient HIS- ID Date of admission, Date of discharge or death or transfer of the patient to another center, Date of the patient fallow up inpatient day bed (normal ward + ICU), Number of normal beds, Number of ICU beds, The patient’s condition at the time of discharge (partial recovery/ discharge with personal consent/referral to another centers /Death) If the patient dies in the hospital: the cause of death must be completed	10
Data registrar profile	Data registrar specifications	M M	Registrar ID, Date of data registration, Registrar name, Registrar surname, Role	5

Patient history such as previous pregnancy and previous delivery, method history of surgery, and comorbidities such as hypertension were classified under the patient history subgroup (as detailed in Table 3).

**Table 3:** Patient history

Main class	Subclass	Mandatory/ Optional data	Data elements	The Number of data elements
Patient History	General health state	M M	Smoking (Number per day) alcohol consumption (Consumption per week) height, Weight BMI (BMI ≥ 18 = slimming / BMI ≥ 25 = normal /BMI ≥ 30 = overweight / BMI ≥ 35 = obese/ BMI ≥ 40 = morbid obesity) Body mass status	6
	Obstetric and gynecological history	M M M M	Menopausal status (Before menopause / menopause) Previous pregnancy history Number of deliveries Previous delivery method (NVD / Cesarean / Both)	4
	Comorbidities	O M M	Taking Anti-coagulant drugs, Previous surgical history (laparotomy /laparoscopy laparotomy and laparoscopy) Disease history (Arterial hypertension / insulin-dependent diabetes/ Type 2 diabetes /Previous or family history of deep vein thrombosis (DVT) / heart disease/ Other	3

In Table 4 Operation data were divided into 2 main groups: general and specialized Surgical information. Surgical approach, medical treatment during surgery, Anesthesia data, operation indications, method, and operation findings were placed in the subgroup of specialized surgical information.

**Table 4:** Operation data

Main class	Subclass	Mandatory/ Optional data	Data elements	The Number of data elements
General information about surgery	General operation data	M M O M	Date of surgery, Surgery time: Duration of operation from the first incision to the last suture (Hours& minutes) Uterine weight (g) without ovaries	4
	Profile of surgeons	M O O	Surgeon’s name Name of the Assistant Surgeon Experience of hysterectomy surgery (less than 10, between 10 and 30, more than 30	3
	Anesthesia data	O M	ASA performance level: (ASA-1^a^, ASA-2^b^, ASA-3^c^, ASA-4^d^,) anesthesia method (General, Spinal, Epidural)	2
Specialized surgical information	Indication method type Preoperative intervention surgical approach	M M M M M M M M O O	Indication: uterine fibroids, Dysfunctional /abnormal uterine bleeding, endometriosis, adenomyosis, Leiomyomas, uterine prolapse, atypical endometrial hyperplasia, infectious disease of the internal genitals, Chronic pelvic pain, Cytological suspicion of endometrial and glandular precancers, Family disp. for Gyn cancer, Surgical method: (abdominal, vaginal, laparoscopic) Type: (total, subtotal, radical) Surgical Approach: abdominal (Total abdominal hysterectomy, Supracervical hysterectomy, radical hysterectomy) Vaginal: (Total, laparoscopically assisted vaginal hysterectomy [ LAVH], Radical vaginal hysterectomy, Other and unspecified vaginal hysterectomy) Laparoscopic: (Total laparoscopic hysterectomy [TLH], vaginal top sutured laparoscopically), Laparoscopic supracervical hysterectomy [LSH], Vaginal-Assisted Laparoscopic Radical Hysterectomy (LRVH)) Vaginal misoprostol / intracervical normal saline infiltration Abdominal incision approach: Paramedian incision, Pfannenstiel incision, Cherney incision, Vertical incision, maylard incision Colpotomy: (Use of unipolar current/ Use of bipolar current/Use of ultrasound/Use of cold scissors - knife) uterus removal management: Removal of uterus in toto(fully), Sharing/ coring with knife/scissors, Use of power morcellator,	9

^a^ without systemic disease, ^b^ Mild systemic disease – ^c^ without functional impairment. ^d^ Severe systemic disease – dysfunction

In addition, a subgroup of technical and surgery approaches has been set up to include Abdominal incision approach, Surgical technique or Suspension as well as other interventions such as Salpingectomy and associated treatments like Enterocele correction. Prophylaxis was also classified as a medical condition during surgery under the category of treatment(Table 5).

**Table 5:** Additional interventions & accompanying treatment

Main class	Subclass	Mandatory/ Optional data	Data elements	The Number of data elements
Peri-operative intervention				
Additional interventions	Additional interventions	M M M M	Salpingectomy: By tomi, By laparoscopy, In case of vaginal surgery: Right TUL1/ Left TUL2 Salpingo-oophorectomy: By tomi or vaginal/ unilat, By tomi or vaginal bilat, By laparoscopy unilat, By laparoscopy bilat Prolapse surgery: Front wall plastic, back wall plastic Colpoperineoplasty	5
Accompanying treatment measures	accompanying actions	M M M	Adherence solution, larger: By tomi, By laparoscopy Incontinence correction: TVT, TOT, other Enteroseel correction	3
	Suspension	O	uterosacral ligament, cardinal ligaments, Apical Suspensions, McCall, Bob Shull, Modified TAIL, Other suspension	1
	blood transfusion	M	Before surgery -------- erythrocyte unit During surgery --------- Red blood cell unit After surgery -------- erythrocyte unit	3
	prophylaxis	M M M M	Preoperative tranexamic acid prophylaxis Antibiotic prophylaxis: Perioperative antibiotics given Prescribing antibiotics during surgery Cefazolin-Cefoxetine-Cefuttan-Cefuroxime-Ampicillin-Sulbactam In penicillin-sensitive patients: Metronidazole + Gentamicin, Metronidazole + Quinolone, Clindamycin + Gentamicin, Clindamycin + quinolone, Clindamycin + Aztero Noam, Metronidazole + Cefazolin, Other Thrombosis prophylaxis: Postoperative Heparin: Rapid and frequent embolization, Mechanical prophylaxis with pneumatic compression intermittent Pharmacological or mechanical prophylaxis Pharmacological and mechanical prophylaxis Unfractionated heparin 5000 Enoxaparin 40 mg, Enoxaparin 40 mg + heparin Pain prophylaxis: NSAID use (mefenamic acid, ibuprofen, naproxen, celecoxib, piroxicam/ acetylsalicylic acid (aspirin)	4

Table 6 lists complications, including complications during surgery, changes in the surgical method, adverse reactions, findings and reoperation during the same hospitalization.

**Table 6:** Peroperative complications

Main class	Subclass	Mandatory/ Optional data	Data elements	The Number of data elements
Peroperative complications	Serious complication during surgery	M M	Bladder damage, Ureteral injury, Intestinal damage More than 1000 ml of surgical leakage Vascular damage: Epigastric vessels / Large vessels (Aorta, vena cava, Iliac) / Other vessels/Complications of anesthesia	2
	Converted perioperatively	M	Converted perioperatively: Laparoscopy to laparotomy, Vaginal to laparotomy	1
	Complications during hospitalization	M M M M M M	Complications cause: Infection: Bladder inflammation, wound or slit infection (requires antibiotics, puncture, evacuation), chest infection, pelvic infection (hematoma or abscess) Urinary tract infection (urine culture above 10^5^) Fever of unknown cause (axillary fever above 38 degrees) Bleeding: bleeding/hematoma of vaginal arch, intraperitoneal bleeding, post-operative bleeding/hematoma Organ damage: Urinary tract damage (ureter, bladder), urogenital fistula, intestinal damage, difficulty in bowel movements, Intestinal obstruction after surgery, prolapse of pelvic organs. Complications of wounds: hernia, abdominal/pelvic abscess, abdominal-pelvic fascia tear, pain, neuropathy Hematoma formation Deep vein thrombosis, Pulmonary embolism Foreign body remains in the abdomen Anemia: (blood transfusion) Other	6

Pathology findings and readmission and repeat surgery up to 30 days after patient discharge, were classified in the follow-up data group (Table 7).

**Table 7:** Follow up data

Main class	Subclass	Mandatory/ Optional data	Data elements	The Number of data elements
Pathology	Pathological results	M	Chronic salpingitis, Dysplasia of the cervix, Metaplasia, Leiomyosarcoma, Cancer of cervix, Endometrioid Adenocarcinoma, Serous adenocarcinoma, Clear cell carcinoma, Endometrial hyperplasia without atypia Benign leiomyoma, Metaplasia, Endometrial hyperplasia with atypia, Endometriosis, Endometritis, Neoplastic ovarian cyst, Adenomyosis, Hydrosalpinx, Other	1
Complications	Complications and readmission	M M M M M M	Date of readmission (maximum 30 days after discharge) Cause of hospitalization, Infection: Inflammation of the bladder Wound or cleft infection requires antibiotics Chest infection, Intra-abdominal infection Urinary tract infection (urine culture above 10^5^) Fever with unknown cause (axillary fever above 38 degrees), Pelvic infection (hematoma or abscess) Hemorrhage: Hemorrhage or wound hematoma Hemorrhage / Hematoma of the vaginal top Intraperitoneal Hemorrhage Injury: Injury of the urinary tract (ureter, bladder), Intestinal injury, Difficulty bowel movements, Postoperative bowel obstruction, Pelvic organ prolapse, urogenital fistula obstruction after hysterectomy, DVT, Anemia: (blood transfusion) Pulmonary embolism, Pain, alone the reason for readmission, Neuropathy	6
	Reoperation procedure in Vaginal cuff rupture	M M	Cause of reoperation: Vaginal cuff dehiscence: Partial superficial defect / Complete wall defect/ Unspecified vaginal obstruction Reoperation procedure: Vaginal cuff rupture repair: Laparotomy Surgery / Laparoscopic surgery /Vaginal surgery	2

**Fig. 1:**
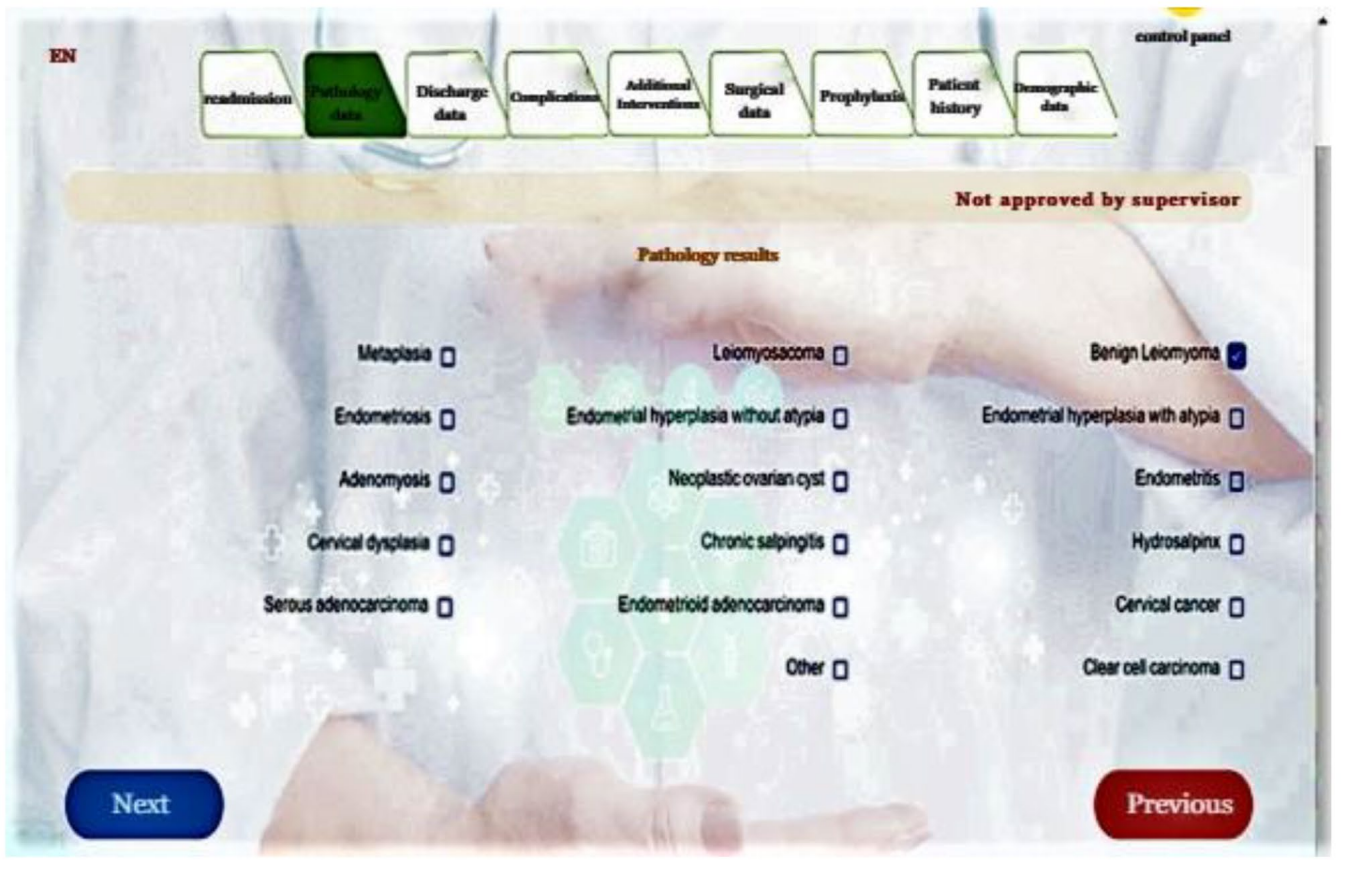
An example of hysterectomy registration system page

In the next phase, web-based Hysterectomy registration software was developed with Visual Studio2019 (Fig. 1).

**Fig. 2:**
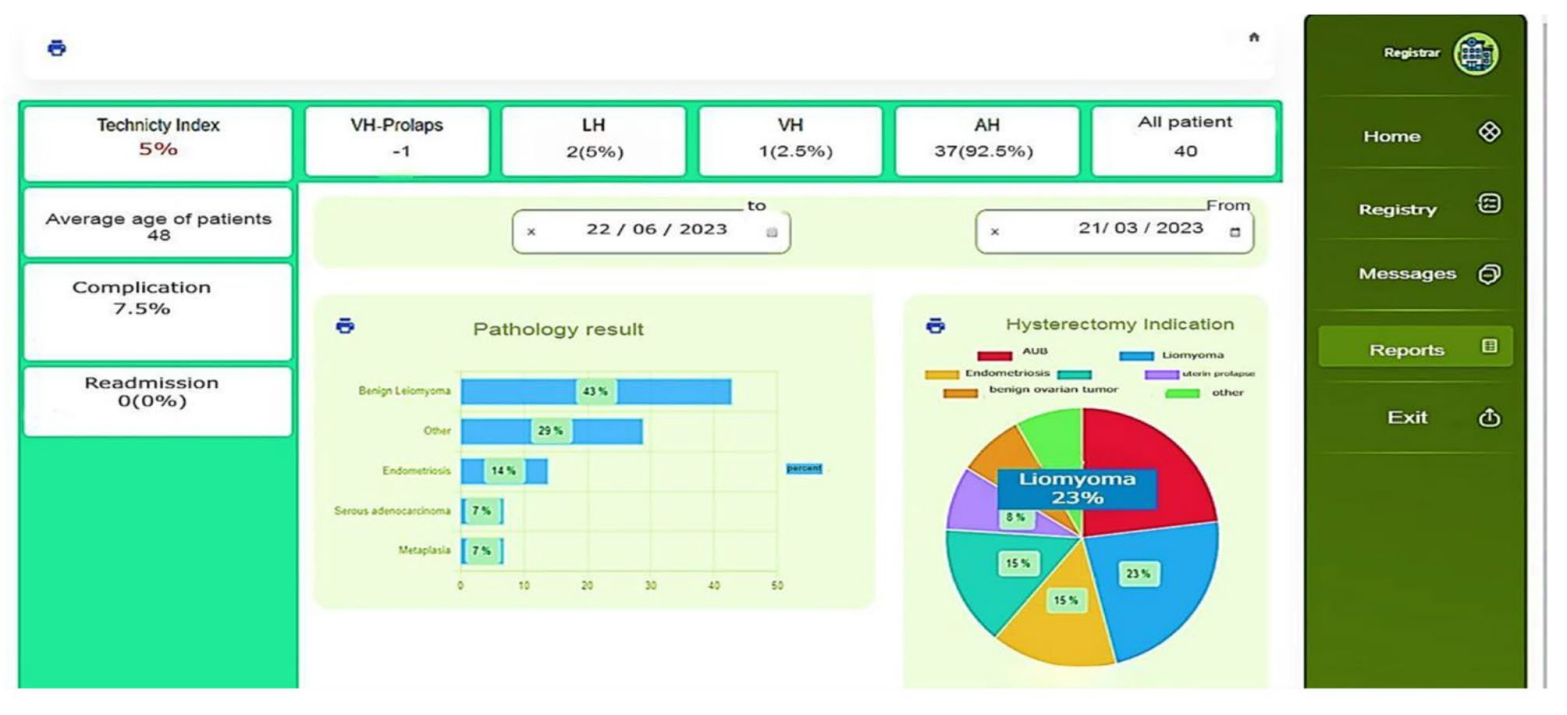
Dashboard of hysterectomy registry system

Automatic calculation of the total number of patients, figures and percentages for each hysterectomy method, an average age as well as a Technicity index in hysterectomies are part of the system dashboard reports. (Fig. 2).

There is an important feature in the hysterectomy registration system, which computes and displays a Technicity index at real-time. The Technicity Index (TI) is a quality metric for hysterectomy, calculated by dividing the number of minimally invasive hysterectomies by the total number of hysterectomies performed during a specific period. A higher TI indicates better quality patient care [25, 26]. A visual diagram also shows the frequency of hysterectomy indications and pathological findings.

Despite the fact that this registry is designed to be used for prospective purposes, during the two months between 21 March and 22 May 2023 patients who were admitted to AlZahra Referral Hospital in Tabriz have had their data retrospectively recorded on paper forms entered into the system with a view to evaluating the effectiveness of the Registry System and ensuring its accuracy.Data from 40 patients have been included in the study and have been successfully registered in the system, out of a total of 54 identified patients. Table 8 presents the initial findings, which indicate that the vast majority (92.5%) of hysterectomies were performed abdominally, with a small percentage (5%) utilizing the LAVH method and an even smaller percentage (2.5%) being performed vaginally. Additionally, over 70% of patients were between the ages of 45 and 55. The most frequently reported pathological findings in our study were leiomyoma, benign ovarian cysts, and adenomyosis. According to the findings of this study, the technicity index in this period of time 5% was reported (Table 8).

**Table 8:** Initial results from registered patient in system

Variables	Frequency	percent
**Patient age:**		
-<40 years 40–55 years 56–65	1 31 9	2.5 77.5 22.5
**Hysterectomy method:**		
Abdominal (AH) total subtotal radical Vaginal (TVH) LAVH Laparoscopic (TLH)	37 33 1 4 1 2 0	92.5 89 2.7 10.8 2.5 5 0

## Discussion

The development of a Minimum Data Set (MDS) is one of the fundamental steps to ensure the standardization of data collection in the disease registration systems. In addition to improving the use of health information, the development and implementation of standardised data sets for clinical registration will support data management and lead to improved quality of care and future interventions. [27, 28]. Also, the use of MDS in clinical studies and research provides opportunities to improve policies and national care programs [29].

The advantage of minimally invasive hysterectomy and the need to reduce the rate of abdominal hysterectomy [30] highlights the importance of designing and implementing a hysterectomy registration system [31]. So far, no comprehensive study has been done regarding the volume of hysterectomy and or the comparison of vaginal, abdominal, and laparoscopic methods about benign hysterectomy in Iran; or At least, We couldn’t identify any study in Iran that was written in English. However, a handful of cross-sectional studies conducted in different regions of Iran indicate a high volume of abdominal hysterectomy for benign indications. The shortage of literature regarding the usage of vaginal or laparoscopic hysterectomy methods in Iran indicates the limited usage availability of these methods in the country. The abdominal hysterectomy approach often results in longer hospital stays, increased discomfort, bleeding, and a greater risk of complications such as wound infections. Therefore, an evaluation and determination of the most appropriate surgery approach in Iran is essential. This study is the first attempt made to develop the core data set for the hysterectomy registries in Iran.

One of the most successful hysterectomy registration systems in the world is the Danish Hysterectomy and hysteroscopy database (DHHD) (Since 2003). All Danish women who have undergone an elective hysterectomy, which is recorded directly by the surgeons involved in the treatment and prospectively [32].

Topsoee et al.‘s in 2016 released the first evaluation of the DHHD registry. Based on this report, the registry has not only met its primary objectives but has also witnessed a rise in the adoption of vaginal and laparoscopic techniques across Denmark. Additionally, there has been a reduction in abdominal hysterectomy rates and surgical complications [33].

In this study, the Danish Hysterectomy and Hysteroscopy Database (DHHD) [34] served as a reference for designing a registry system, even though he used methods were different. However, several clinical data elements were common or had high similarity, also there was an overlap between the current core data set and data elements in the Finnish benign hysterectomy cohort study [35]. Clinical guidelines and Research in the field of hysterectomy were other data used in this study. Management data elements and demographic information were also selected based on existing standards and studies in Iran. Finally, a web-based registration system, with Remote access capability was developed based on the selected dataset and approved by the expert’s group.

One significant characteristic of an electronic registration system for hysterectomy is the ability to dynamically display and calculate various factors such as technicity index, volume, method of hysterectomy, and pathology results. This allows for easy monitoring and reporting of the system over a specific period of time and can aid in the decision-making process for community health policymakers.

Based on the initial reports of this system and consistent with the findings of conducted studies, it seems unlike many countries with greater financial medical support, the utilization of vaginal hysterectomy is relatively restricted compared to the laparoscopic approach in Iran. Of course, experts in this field should comment on the reasons for this.

ased on the research by Einarsson et al.,, gynecologists encounter various challenges when performing vaginal hysterectomies (VH). These challenges include technical intricacies, potential complications, and increased workload. Similarly, gynecologists face obstacles when performing laparoscopic hysterectomies (LH), such as suboptimal training during residency, technical complexities, limited surgical experience, and prolonged operation times [36].

The technicity index in this study 5% was reported. Further research and analysis are necessary to identify the underlying factors contributing to this low index. On otherwise, these findings highlight the need for further research in Iran and professional development to support gynecologists in managing these challenges and delivering optimal patient outcomes.

## Conclusion

Creating a hysterectomy registration system is the initial stride toward identifying and registering hysterectomy candidate patients. this system capture information about the procedure techniques, and associated complications. In Iran, this registry can serve as a valuable resource for assessing the quality of care delivered and the distribution of clinical measures.

The limited use of minimally invasive procedures is a matter of concern and requires further investigation and action. Since there is a dearth of research in this domain, It is recommended to establish a nationwide hysterectomy registration system to identify rates, techniques, and associated hysterectomy complications. This, in turn, can provide healthcare providers with valuable insights into the effectiveness of current practices, and allow for better decision-making on future policy making.

## Data Availability

The data that support the findings of this study are available from al-Zahra Educational, Research and Treatment Center, but restrictions apply to the availability of these data, which were used under license for the current study, and so are not publicly available. Data are however available from the authors upon reasonable request and with permission of al-Zahra center.
